# Causes of nutrition deficit during immediate postoperative period after free flap surgery for cancer of the head and neck

**DOI:** 10.1007/s00405-020-06206-1

**Published:** 2020-07-14

**Authors:** Juho Nurkkala, Sanna Lahtinen, Timo Kaakinen, Merja Vakkala, Janne Liisanantti

**Affiliations:** Department of Anesthesiology, Medical Research Centre and Research Group of Surgery, Anesthesia and Intensive Care, University of Oulu, Oulu University Hospital, P.O. Box 21, 90029 Oulu, Finland

**Keywords:** Head and neck cancer, Free flap, Nutrition, Nutrition support, Enteral nutrition, Parenteral nutrition

## Abstract

**Purpose:**

The aim of the present of study was to examine nutrition deficit during the immediate postoperative in-hospital period following free flap surgery for cancer of the head and neck (HNC). Underfeeding and malnutrition are known to be associated with impaired short- and long-time recovery after major surgery.

**Methods:**

This single-center retrospective cohort study included 218 HNC patients who underwent free flap surgery in Oulu University Hospital, Finland between the years 2008 and 2018. Nutrition delivery methods, the adequacy of nutrition and complication rates were evaluated during the first 10 postoperative days.

**Results:**

A total of 131 (60.1%) patients reached nutritional adequacy of 60% of calculated individual demand during the follow-up period. According to multivariate analysis, nutrition inadequacy was associated with higher ideal body weight (OR 1.11 [1.04–1.20]), whereas adequate nutrition was associated with higher number of days with oral food intake (OR 0.79 [0.67–0.93]).

**Conclusion:**

Inadequate nutrition is common after HNC free flap surgery. The present results suggest that more adequate nutrition delivery might be obtained by the early initiation of oral food intake and close monitoring of nutrition support.

## Introduction

Among patients undergoing major surgical operations, preoperative malnutrition and postoperative underfeeding are recognized risk factors for postoperative complications such as prolonged hospital length of stay (LOS), higher incidence of infectious complications, impaired physical recovery and increased morbidity [1]. It is essential to maintain adequate nutrition after surgical procedures as it improves wound healing. Underfeeding is known to impede normal wound recovery process and is related to wound infections and impaired wound tensile strength [2]. Previous studies contemplating the adequacy of postoperative nutrition have been conducted mainly after gastrointestinal, orthopedic and thoracic surgery [1, 3–6]. There is a lack of studies focusing on the nutrition adequacy after free flap reconstruction of the tumor resections for the cancer of the head and neck (HNC).

Median hospital LOS after free flap surgery for HNC is more than week [7–9]. In-hospital nutrition plays a significant role in the early recovery in this patient group [10]. Although HNC free flap surgery may have a substantial impact on normal eating, chewing and swallowing, there is a lack of studies contemplating the adequacy of nutrition during the immediate postoperative period. Studies concerning the postoperative nutrition of hospitalized HNC patients have mostly been focusing on the preceding prevalence of malnutrition [11, 12] and optimal initiation of oral food intake such as in laryngectomized patients in terms of pharyngocutaneus fistula formation [13–15].

The aim of the present study was to evaluate the adequacy of nutrition after free flap surgery for HNC during the immediate in-hospital recovery and to discover factors associated with inadequate nutrition delivery.

## Materials and methods

This retrospective longitudinal cohort study was conducted in Oulu University Hospital in Oulu (OUH), Finland. The study protocol was accepted by the hospital administration (208/2015 and 239/2016). Following the local policy, no statement from the ethics committee was obtained due to the retrospective study design.

### Patients

The study population consisted of all patients undergone HNC free flap surgery in Oulu University Hospital between the years 2008 and 2018. During the study period, a total of 247 free flap operations were performed. 29 cases were excluded from the analysis due to incomplete data leaving a total of 218 cases. Since the median hospital length of stay (LOS) is more than 7 days in this patient group, we included the first 10 postoperative in-hospital days in the analysis [7, 9].

### Nutrition delivery

The ideal body weight (IBW) was calculated for each patient using the Devine formula for men and the Robinson formula for women [11]. The energy demand during the study period was calculated individually for each patient using the ideal weight and the estimate of 30 kcal/kg/day, which was derived from both the ESPEN guidelines for surgical patients and from the national nutritional guidelines of the UK for HNC patients [1, 10]. Current literature is lacking studies focusing on the adequacy of postoperative nutrition of HNC free flap patients. We selected a cut-off value of 60% of calculated energy demand to mark adequate nutrition in the present study. The cut-off value of 60% has been used in an intensive care setting to depict the difference between satisfactory nutrition and malnutrition [16, 17]. The screening of malnutrition was performed with a modified nutrition-related index (NRI) presented by Parhar et al. [11]. NRI was calculated by the following formula: [1.519 × serum albumin(g/l)] + [41.7 × (mass/IBW(kg))]. Patients with NRI less than 97.5 were considered as preoperatively malnourished. The total amount of consumed intravenous 5% dextrose, PN (parenteral nutrition), EN (enteral nutrition) and oral food intake was calculated by adding all delivered calories during the follow-up time and dividing the sum by the number of follow-up days. Oral food intake calories were calculated from the patient records by inspecting the daily food consumption in milliliters and approximating the daily content of calories based on the average hospital diet (1800 kcal/day in OUH). In the present study, the nutrition support consisted 5% dextrose, PN and EN calories. The loss of appetite, gastric pain and nausea were recorded when the patient refused oral or enteral nutrition at least once due to these reasons during the follow-up time. The study group with < 60% of calculated energy demand is described below as “study group low”, and the study group with > 60% of calculated energy demand is described below as “study group adequate” for clarity issues.

### Postoperative complications

Since perioperative underfeeding and malnutrition are known to have an impact on postoperative complications and outcome [1], we aimed to evaluate rates of complications in the present study. Complications were classified as medical and surgical and were recorded as in our previous study [18].

### Statistical analysis

Statistical analysis was performed with IBM SPSS Statistics 25 software (IBM SPSS Statistics for Windows, Version 25.0, Armonk, NY, USA). Categorical data are expressed as numbers (*n*) and percentages (%) whereas continuous data are expressed as medians and 25th–75th percentiles [25th–75th PCT]. Categorical data were tested using the Pearson’s chi square and the continuous variables were tested using the non-parametric Mann–Whitney test. Two-tailed *P *value was considered statistically significant when it was below 0.05. The logistic regression analysis was used to calculate OR for not reaching the 60% nutrition adequacy cut-off value. Continuous and categorial variables with univariate significance < 0.1 as well as age and gender were included one by one using the enter method. The factors with *P* value < 0.05 were kept in the model, as well as those with significant impact on the log-likelihood function.

## Results

There were a total of 131 (60.1%) patients who achieved the cut-off value of nutritional adequacy in the study group adequate. Patients in the study group low were more often males, had a higher IBW and a higher rate of smoking and alcohol abuse and they had a longer hospital LOS (Table 1). Among the 115 patients with larynx or oral cavity/tongue tumor, a total of 54 (46.9%) were in group low in contrast to 33 of 103 (32.0%, *P* = 0.04) in other tumors.

**Table 1:** Patient demographics

	Group adequate (*N* = 131)	Group low (*N* = 87)	*P* value
Male gender	56 (41.2)	65 (74.7)	< 0.001
Age, years	67.0 [57.0–75.0]	63.0 [57.0–73.0]	0.172
Ideal body weight (kg)	59.5 [52.3–66.8]	68.7 [59.6–75.0]	< 0.001
BMI	24.4 [21.6–27.9]	23.9 [20.0–27.3]	0.147
Preoperatively malnourished	20 (15.3)	18 (20.7)	0.459
Smoking	49 (37.4)	45 (51.7)	0.050
Alcohol	31 (23.7)	34 (39.1)	0.021
Tumor			
Oral cavity/tongue	53 (40.5)	40 (46.0)	0.072
Maxilla	13 (9.9)	10 (11.5)	
Mandible	24 (18.3)	12 (13.8)	
Larynx	8 (6.1)	14 (16.1)	
Skin melanoma	12 (9.2)	6 (6.9)	
Buccal mucosa	15 (11.5)	5 (5.7)	
Parotid gland	5 (3.8)	0 (0.0)	
Lymphoma	1 (0.8)	0 (0.0)	
Stage T1-2	33 (25.2)	26 (29.9)	0.638
Stage T3-4	73 (55.7)	48 (55.2)	
Hospital LOS (days)	10 [8–14]	13 [8–16]	0.029

*BMI* body mass index, *LOS* length of stay. Values are numbers (percentage) or medians [25th–75th percentiles].

**Fig. 1:**
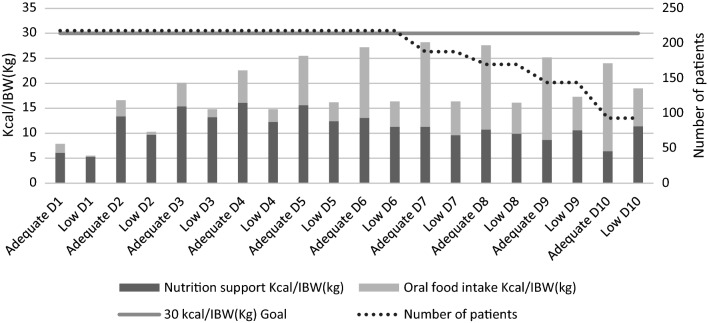
The mean daily delivery of nutrition support and oral food intake. The values are presented for the group adequate and for the group low as the administered calories divided by ideal body weight (IBW in kg’s)

The patients in the study group adequate had a lower calculated daily energy demand, higher administration of enteral calories, earlier initiation of oral food intake and a higher number of days with oral food intake. Patients in the study group low had more often gastric pain (Table 2). In the study group, adequate oral food intake increased overall nutrition adequacy. Nutrition adequacy improved during first seven days but started to decrease as the number of discharged patients increased (Fig. 1).

**Table 2:** Nutritional characteristics of the patients receiving either ≥ 60% or < 60% of calculated energy demand

	Group adequate (*N* = 131)	Group low (*N* = 87)	*P* value
Calculated daily energy demand (kcal/d)	1784 [1569–2005]	2060 [1788–2250]	< 0.001
Administered daily 5% dextrose (kcal)	97 [56–140]	91 [43–154]	0.773
Administered daily PN (kcal)	200 [97–280]	174 [70–288]	0.501
Administered daily EN (kcal)	344 [115–630]	374 [178–573]	0.822
Administered daily oral food intake (kcal)	675 [315–900]	180 [0–550]	< 0.001
First day of oral food intake	5 [3–7]	6 [5–8]	< 0.001
Days of oral food intake	6 [4–8]	5 [3–6]	0.001
Loss of appetite	21 (16.0)	14 (16.1)	> 0.9
Gastric pain	12 (9.2)	19 (21.8)	0.010
Nausea	30 (22.9)	16 (18.4)	0.499

*PN* parenteral nutrition, *EN* enteral nutrition. Values are numbers (percentage) or medians [25th–75th percentiles]

In the surgical characteristics, the patients in the study group low had more often tracheostomy and bilateral neck dissection (Table 3).

**Table 3:** Surgical features of the patients receiving either ≥ 60% or < 60% of calculated energy demand

	Group adequate (*N* = 131)	Group low (*N* = 87)	*P* value
Tracheostomy	96 (73.3)	74 (85.1)	0.046
Bilateral neck dissection	14 (10.7)	19 (21.8)	0.016
Unilateral neck dissection	87 (66.4)	42 (48.3)	
No neck dissection	30 (22.9)	26 (29.9)	
Flap type			
RFA	47 (35.9)	30 (34.5)	0.560
ALT	46 (35.1)	23 (26.4)	
LD	3 (2.3)	1 (1.1)	
Scapula	6 (4.6)	3 (3.4)	
Fibula	18 (13.7)	15 (17.2)	
Lateral arm	6 (4.6)	7 (8.0)	
Other	5 (3.8)	7 (8.0)	
Intraoperative blood loss (ml)	600 [350–1000]	550 [350–950]	0.474
Intraoperative infused fluids (ml)	5910 [4450–7870]	5890 [4400–7330]	0.514
Intraoperative duration of MAP < 65 (min)	148 [50–256]	129 [42–336]	0.752
Length of surgery (min)	495 [426–620]	541 [440–621]	0.259

*RFA*  radial forearm flap, *ALT* anterolateral thigh flap, *LD* latissimus dorsi flap, *MAP*  mean arterial pressure. Values are numbers (percentage) or medians [25th–75th percentiles]

The patients in the study group low had more often postoperative surgical site infections, although there was no difference between the groups in the onset of infection (3 [2–10] vs 6 [2–11], *P* = 0.488). There was no difference in the incidence of partial or total loss of flap but the patients in the study group low suffered a loss of flap earlier than in the study group adequate (3 [2–20] vs 10 [6–13], *P* = 0.050). Overall postoperative surgical complications were more frequent among patients in the study group low. There was no difference in the incidence or distribution of medical complications between the study groups (Table 4).

**Table 4:** Postoperative complications

	Group adequate (*N* = 131)	Group low (*N* = 87)	*P* value
Surgical complications	54 (41.2)	49 (56.3)	0.037
Surgical site infection	21 (17.3)	29 (30.7)	0.003
Onset of infection (d)	6 [2–11]	3 [2–10]	0.488
Surgical site hematoma	23 (17.6)	16 (18.4)	> 0.9
Onset of hematoma (d)	2 [1–5]	4 [2–7]	0.128
Reoperation	36 (27.5)	33 (37.9)	0.104
Onset of reoperation (d)	5[1–9]	2 [1–9]	0.875
Partial or total flap loss	9 (6.9)	9 (10.3)	0.452
Onset of partial or total flap loss (d)	10 [6–13]	3 [2–20]	0.050
Medical complications	36 (25.2)	24 (26.4)	> 0.9
Pneumonia	21 (16.0)	18 (20.7)	0.471
Onset of pneumonia (d)	4 [2–7]	7 [3–10]	0.140
Sepsis	5 (3.8)	2 (2.3)	0.705
Sepsis onset days	2,7,8,8,21	1,13	
Pulmonary edema	9 (6.9)	3 (3.4)	0.370
Pulmonary edema onset days	1,3,4,6,6,7,8,9,18	4,7,9	
Acute myocardial infarction	0 (0.0)	2 (2.3)	0.158
Acute myocardial infarction onset days	–	1,3	
Deep venous thrombosis, pulmonary embolism, stroke	4 (3,1)	2 (2,3)	0.732
Deep venous thrombosis, pulmonary embolism, stroke onset days	2,3,5,11	2,9	

Values are numbers (percentage) or medians [25th–75th percentiles]. Sepsis, pulmonary edema, acute myocardial infarction and deep venous thrombosis/pulmonary embolism/stroke onset days are reported without percentiles due to small count of cases

In the logistic regression analysis, a higher IBW was associated with a failure to reach nutritional targets whereas a higher number of days with oral food intake was associated with a decreased OR for impaired nutrition delivery (Table 5).

**Table 5:** OR and 95% confidence intervals for impaired nutrition delivery according to the logistic regression analysis

	OR (95% CI)	*P* value
Male gender	0.52 (0.14–1.84)	0.307
Age	1.01 (0.98–1.04)	0.468
Ideal body weight	1.11 (1.04–1.20)	0.002
Surgical complications	0.84 (0.40–1.76)	0.640
Days with oral food intake	0.79 (0.67–0.93)	0.005

## Discussion

For our understanding, this is the first study evaluating postoperative in hospital nutrition adequacy after HNC free flap surgery. The main finding of the present study was that inadequate nutrition delivery was common in patients with HNC undergoing free flap surgery. Approximately, 40% of patients did not achieve the cut-off value of 60% of calculated energy demand during the first 10 postoperative days. Our results suggests that an early initiation of oral food intake significantly improved nutrition delivery. Enteral or parenteral nutrition support alone often did not provide adequate nutrition delivery for patients without oral intake. Patients with lower IBW were more likely to reach a sufficient level of calories when compared patients with higher IBW indicating that individual demand of energy was not always taken into consideration. Patients whose operation affected oral cavity or larynx had lower nutritional status than other subgroups. Patients with postoperative surgical complications were associated with nutrition inadequacy, although the difference was not seen in the logistic regression analysis.

According to our results, an early initiation of oral food intake improved nutrition delivery considerably. Furthermore, oral food intake was a more efficient way to deliver nutrition compared to nutrition support, although calculated caloric demand was never achieved with any nutrition delivery method. Both the ESPEN guidelines and the national nutritional guidelines of the United Kingdom (UK) for HNC patients promote early postoperative oral feeding or nutrition support in this patient group [1, 10]. It is previously known that HNC patients undergone free flap surgery are rarely able to eat normally immediately after the operation [1, 10]. Common reasons for impaired oral food intake after HNC free flap surgery are dysphagia and swallowing-related pain [7, 18]. Due to anatomic and physiological reasons, swallowing may be permanently deteriorated after free flap surgery for HNC [19]. HNC surgery may also cause significant oral cavity or upper gastrointestinal tract swelling during first postoperative days inhibiting oral food intake [1]. It is also reported recently by Lilja et al. that HNC free flap surgery affecting oral cavity may impair taste sensation which affects negatively oral food intake [20]. Moreover, growing HNC tumor may develop upper gastrointestinal tract obstruction which causes difficulties in swallowing and furthermore induces patients to eat less than without the illness [11].

In the past, surgeons used to delay the initiation of oral food intake among HNC patients with free flap surgery affecting the oral cavity for avoiding adverse outcomes in wound healing and reducing the risk of dehiscence. Recent studies have shown that early oral food intake after surgery affecting the oral cavity is safe [8, 21]. For example, many studies from recent years have shown that early oral food intake does not increase pharyngocutaneus fistula formation. Nowadays early oral food intake is recommended also for patients with total laryngectomy [10, 13–15]. According to the present study, it seems that efforts should be made to initiate oral food intake as soon as possible after the operation to obtain adequate nutrition delivery. However, due to reasons mentioned above, oral food is rarely given during the very first postoperative days and in the previous literature “early oral intake after HNC surgery” is generally determined as oral nutrition given before or during the fifth postoperative day [14, 21] which in this study was the median oral food initiation day in the study group adequate.

The results of the present study suggest that nutrition support did not provide sufficient intake of calories for patients whose oral food intake was inadequate. Patients unable to eat could benefit from individual nutrition support to enhance their overall nutrition status. In the intensive care setting, it has been reported that nutrition adequacy can be as low as 30% of calculated demand when feeding is conducted solely by nutrition support [22]. The impact of nutrition support has become more evident in the intensive care by the introduction of nutrition support protocols, inspecting the patient’s nutritional status twice a day and by including a routine dietician consultation for every patient [16, 23]. These methods could also be utilized in HNC patients to enhance nutrition support.

The patients with tracheostomy had a greater risk for postoperative undernutrition in the univariate model. The negative effect of tracheostomy to nutrition delivery was an expected finding since the procedure diminishes tolerance to oral food intake which was the most dominant nutrition delivery method in the present study [24]. Bartella et al. reported recently strong statistically significant relation between impaired swallowing function and prolonged timing of decannulation [25]. Moreover, Goetz C et al. recommended that the duration of temporary tracheostomy in HNC patients should be as short as possible to reduce associated complications [26]. Considering the nutritional aspect, a similar conclusion can be made based on our results. In the present study, bilateral neck dissection was a risk factor for impaired nutrition adequacy in the univariate model. It has been previously reported that bilateral neck dissection has a negative impact on swallowing function after HNC free flap surgery [19], which might explain the association between a poor nutrition adequacy and bilateral neck dissection.

The occurrence of surgical complications was more frequent among patients with inadequate nutrition delivery. Postoperative surgical site infections were more frequent in patients with impaired nutrition adequacy in the present study. It has been reported that postoperative malnutrition in surgical and intensive care patients predisposes to infectious complications [1, 2, 10, 16, 23]. On the other hand, it is known that postoperative surgical site infections are a risk factor to reoperations [9], which may predispose to malnutrition caused by preoperative fasting. In the present study, however, there was no difference in the onset of infection between the study groups. There was also no statistically significant difference in the incidence rates of partial or total loss of flaps between the study groups but patients with a lower nutrition adequacy had flap losses in an earlier phase of their recovery. Partial or total loss of flap may cause delay in initiation of oral food intake due to preoperative fasting before reoperations. Also, uncertainty of the healing status of wounds affecting oral cavity may cause surgeon to postpone initiation of oral nutrition. Group adequate had their loss of flaps later which may explain why their nutrition status was higher during present follow-up. No difference was found in other subgroups of surgical or medical complications. Due to the retrospective nature of the study and relatively short follow-up time, a causal relationship between inadequate nutrition delivery and the occurrence of postoperative complications remains obscure.

### Clinical significance

The negative impact of postoperative malnutrition on both short- and long-term recovery of surgical patients has been widely acknowledged and, therefore, pursuing adequate nutrition delivery is desirable [1, 10]. The results of present study add to the knowledge that reaching sufficient nutrition for HNC free flap patients postoperatively is beneficial. Based on our results, efforts should be made to obtain sufficient nutrition delivery and focus should be turned to early oral food intake. This could be attained by adequate pain medication and by utilizing consultation of a dietician and a physiotherapist in the immediate recovery phase [10]. For patients whose oral food intake is insufficient, nutrition support delivery could be increased by using specified nutrition protocols and by inspecting the nutritional status twice a day [1, 23]. A multidisciplinary team (surgeon, physiotherapist, dietician, trained nurse) could be very useful in establishing and enhancing individual nutritional support for patients after HNC free flap surgery as well as after any major surgery [10]. Further research, preferably in a prospective setting, should be conducted to enlighten the possible connection between these proposed means and adequate nutritional delivery among HNC free flap patients.

## Limitations

This study has some limitations. The study population is quite heterogenous since we included all HNC free flap patients into the analysis. Because the impact of free flap operations on nutritional delivery may vary significantly between the patients, the heterogenous study population may lead to different nutritional outcomes between different subgroups. However, by including all subgroups to the present analysis, our study design mimics actual surgical ward setting quite accurately. In the present study, it was not possible to analyze nutrition prescription rate since in our hospital, nutrition support is nurse driven and formal prescriptions are rarely done by the attending physician or dietician. The reasons why oral food delivery was not initiated during the first or second postoperative days in this cohort remains partly obscure. Initiation judgement of oral food delivery is ultimately done based by attending surgeon’s evaluation. Problems in surgical area such as swelling, pain, poor tissue healing as well as attending surgeon’s customs may postpone oral food initiation. However, to analyze this phenomenon in detail, it would require a study preferably in a prospective setting. The retrospective study design and a relatively short follow-up period of the study causes limitations when inspecting causal relationship between inadequate nutrition delivery and the rate of complications. However, based on our findings, it can be concluded that surgical complications are associated with inadequate nutrition delivery. Finally, the aim of the present study was to analyze nutrition adequacy and factors related to inadequate nutritional delivery, not to investigate complications associated underfeeding.

## Conclusion

Underfeeding is common within HNC free flap patients during the immediate postoperative period. Early oral food intake was associated with increased nutrition delivery and nutrition support often failed to provide enough nutrition to cope with patient’s energy demand. The results of the present study suggest that oral food intake is the most effective way to commence nutrition in HNC free flap patients postoperatively. Guidelines contemplating the issue promote early nutrition support for patients unable to ingest food [1, 10] but it seems that nutrition support alone is prone to be inadequate in this patient group. The findings of the present study should be taken into the account when contemplating the postoperative nutrition of HNC free flap patients.

## Data Availability

Not applicable.
